# Movement efficiency in taekwondo side kick (*Yop Chagi*): a kinematic comparison between novice and experienced practitioners

**DOI:** 10.3389/fphys.2025.1708748

**Published:** 2026-01-08

**Authors:** Rahmat Hidayat, Xianzhi Jin, Chengji Dou, Benyao Yang

**Affiliations:** Department of Fundamental Education, Qilu Institute of Technology, Shandong, Jinan, China

**Keywords:** biomechanics, martial arts, motion analysis, motor skills, performance optimization, skill acquisition

## Abstract

**Background:**

This study aimed to kinematically compare the Yop Chagi execution between novice and experienced university Taekwondo practitioners, focusing on movement efficiency.

**Methods:**

Forty university students were allocated into two groups: Novice (n = 20; ≤6 months experience) and Experienced (n = 20; ≥3 years of competitive experience). Participants performed maximal effort side kicks targeting a pad at trochanter height. Movement was captured using two high-speed cameras (120 Hz). Kinematic variables included hip and knee joint angles at peak flexion/extension, peak angular velocities, linear velocity of the foot, and total kick execution time. Between-group differences were analysed using independent t-tests or Mann-Whitney U tests, with effect sizes (Cohen’s d) reported.

**Results:**

The experienced group demonstrated significantly larger hip abduction and knee flexion angles during the chamber phase (*p* < 0.01, d > 0.8), and greater knee extension at the point of impact (*p* < 0.01, d = 1.2) compared to novices. The experienced group also exhibited superior performance in peak hip and knee angular velocities (*p* < 0.01, d > 1.5), resulting in a 28% higher peak linear velocity of the foot (*p* < 0.001, d = 2.1). Furthermore, the total execution time was significantly shorter for the experienced group (*p* < 0.01, d = 1.4).

**Conclusion:**

Experienced practitioners execute the Yop Chagi with a more efficient kinematic pattern, characterized by a larger range of motion, faster segmental velocities, and reduced execution time. These findings suggest that long-term training optimizes the proximal-to-distal kinematic sequence, enhancing kick performance.

## Research background

Taekwondo is one of the most widely practiced martial arts worldwide, and kicking techniques represent its most distinctive feature (Koh et al., 2004; Mingming, 2013). The effectiveness of taekwondo performance largely depends on the ability of athletes to execute kicks with high speed, power, and precision. Among various techniques, the side kick (Yop Chagi) is considered both a fundamental and strategic technique, frequently utilized in training as well as competition (Yao, 2023; Yunpeng, 2014). Observational studies of competitive matches indicate that side-kick-type techniques account for approximately 6%–13% of total kicking actions, highlighting Yop Chagi as a tactically relevant technique frequently employed in competition (Huang et al., 2025; Ko et al., 2025; Preuschl et al., 2016) This evidence emphasizes the practical importance of side kick performance for both offensive and defensive strategies.

The quality of its execution is determined by efficient coordination of body segments, leg velocity, and postural control (Barnamehei et al., 2020; Cheng et al., 2015). In the field of sports biomechanics, kinematic analysis provides a valuable approach to understanding how movements are performed without reference to the forces that cause them. Such analysis encompasses variables including joint angles, angular velocity, phase duration, and trajectory of motion (Diniz et al., 2021; Jakubiak and Saunders, 2008; Tsung et al., 2018). Through this lens, it becomes possible to identify differences in movement patterns between novice and experienced practitioners, reflecting the degree of motor skill development achieved through prolonged practice and training (Góra et al., 2025; Jung and Park, 2018; Kwan Kim et al., 2011).

Previous studies have examined the biomechanics of taekwondo kicks, showing that experienced athletes demonstrate more efficient hip and knee coordination and achieve higher kicking speeds than novices, particularly in roundhouse (Dollyo Chagi) and front kicks (Ap Chagi) (Estevan et al., 2011; Falco et al., 2011; Suppiah et al., 2018; Wąsik, 2011). However, research specifically on the side kick (Yop Chagi) is limited, and few studies have addressed performance differences between novice and experienced university athletes. This is a critical gap, as university populations encompass both beginners learning fundamental motor skills and advanced students with competitive experience. Investigating these differences is essential to understand how experience and long-term practice influence movement efficiency in an educational and athletic context.

This study provides a novel contribution by analyzing the kinematics of the side kick (Yop Chagi) in novice and experienced university taekwondo practitioners. Unlike previous research focused on other kicks or elite athletes, this study examines a commonly used yet underexplored technique within an educational context. By highlighting differences in movement efficiency and coordination, the findings offer actionable insights for coaches and physical education instructors, while simultaneously advancing biomechanical knowledge and addressing a significant gap in the literature on Yop Chagi.

## Participants

A total of forty participants were included in the study (Novice = 20; Experienced = 20). The novice group comprised 12 males and 8 females, while the experienced group consisted of 13 males and 7 females. No significant differences were observed in sex distribution between groups (χ^2^ = 0.10, *p* = 0.75). Participants characteristics are summarized in Table 1.

**TABLE 1:** Participant characteristics (mean ± SD).

Variable	Novice		Experienced	
	Male (n = 12)	Female (n = 8)	Male (n = 13)	Female (n = 7)
Age (years)	20.5 ± 0.7	20.3 ± 0.6	21.2 ± 0.8	21.0 ± 0.7
Height (cm)	173.1 ± 5.9	166.2 ± 5.2	174.2 ± 5.8	168.3 ± 5.0
Body Mass (kg)	68.4 ± 6.9	61.5 ± 6.2	69.2 ± 6.7	63.4 ± 6.0

Inclusion criteria (Novice): (i) enrolled students with ≤6 months of formal taekwondo instruction; (ii) no prior competition experience; (iii) training frequency ≤1 session·week in the past 6 months. Inclusion criteria (Experienced): (i) ≥3 consecutive years of formal taekwondo practice; (ii) current or former competitive experience at university/regional level or higher; (iii) training frequency ≥3 sessions·week in the past 6 months. General criteria (both groups): age 18–25 years; right-foot dominant (self-report ball-kicking test); normal or corrected vision; free of lower-limb injury, surgery, or pain in the past 6 months; no neurological or vestibular disorders. An *a priori* power analysis (G*Power 3.1, two-tailed independent t-test, α = 0.05) indicated that a total sample of 40 provides ≥0.80 power to detect medium-to-large between-group effects (d ≈ 0.65–0.80). Participants provided written informed consent. The study protocol was approved by the institutional ethics committee and adhered to the Declaration of Helsinki.

## Protocol

**FIGURE 1:**
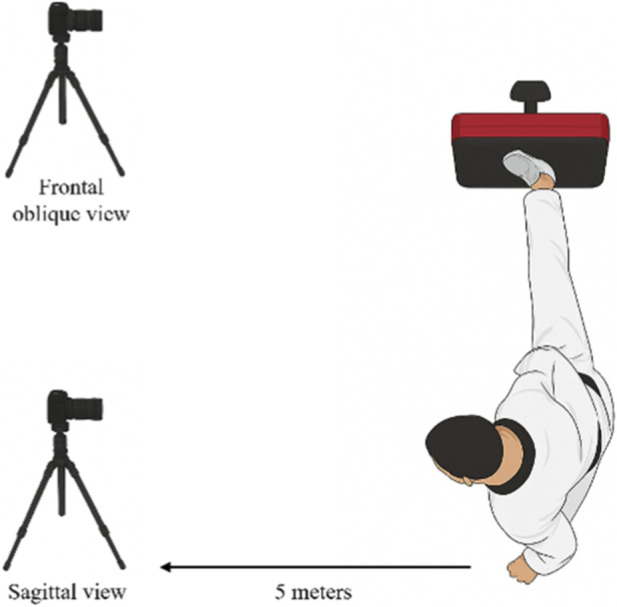
Illustrates the camera layout employed during the motion analysis.

The kinematic analysis of the Yop Chagi technique was conducted using the Dartfish system. Data collection took place in an indoor gym in Semarang, Central Java, Indonesia, with controlled temperature (22 °C–24 °C). Participants attended one familiarization session prior to testing to practice the technique and become accustomed to the equipment. They wore standardized sports clothing and refrained from consuming caffeine, alcohol, or medications that could affect performance within 24 h before testing. A standardized 5-min warm-up protocol, including dynamic stretching and submaximal kicking drills, was performed before trial. Video data were collected using two high-speed digital cameras (120 frames per second, 1080p resolution) positioned at standardized locations. The primary camera was placed 5 m laterally from the participant at a height of approximately 1.0 m (hip height) to capture the sagittal plane of motion, while the secondary camera was positioned 5 m from the participant at a 45° frontal-oblique angle and a height of approximately 1.0 m to verify movement trajectories and minimize parallax errors. Figure 1 presents a schematic illustration of the camera setup to clarify the relative lateral and frontal positions; however, all camera distances, heights, and angles were precisely measured and consistently applied during the actual data collection. Both cameras were calibrated using a two-dimensional reference frame prior to testing.

**FIGURE 2:**
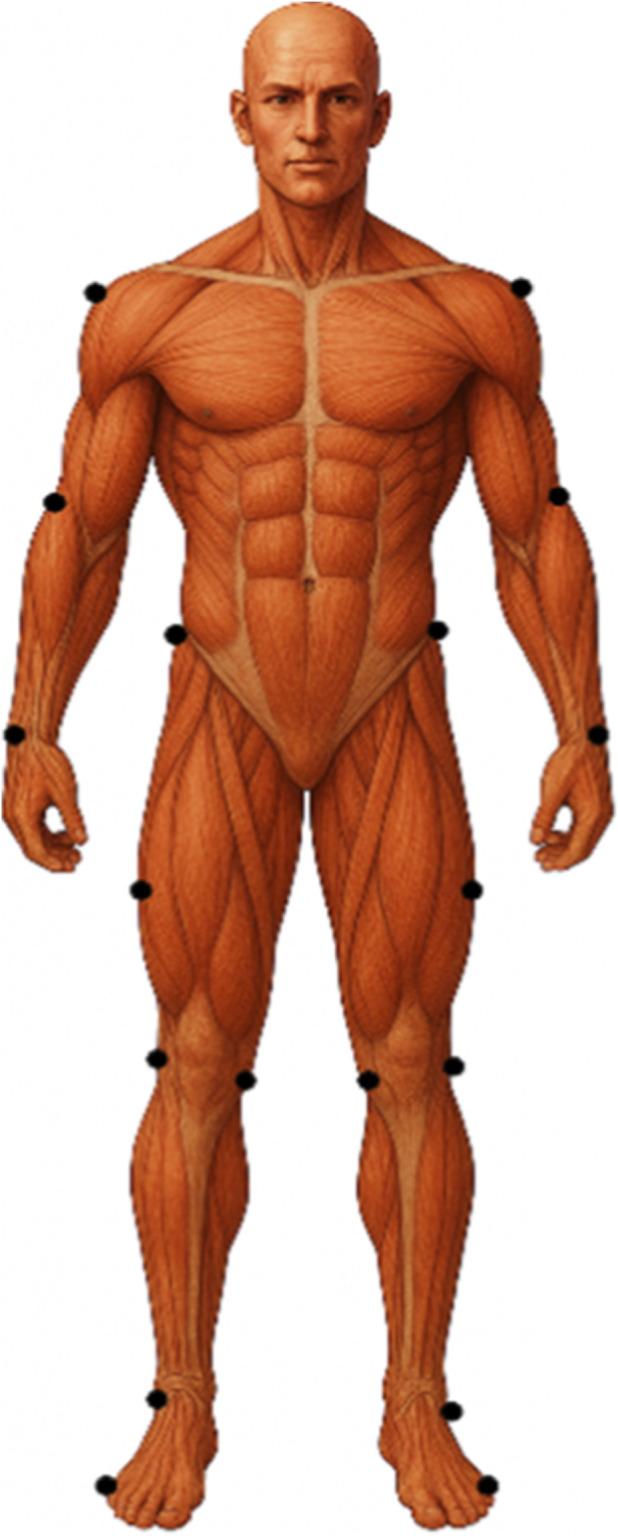
Marker placement on anatomical landmarks for kinematic analysis of the Taekwondo Side Kick (Yop Chagi) technique.

Participants were instructed to perform eight maximal side kicks (Yop Chagi) using their dominant leg, targeting a kicking pad adjusted to the height of the greater trochanter to ensure consistent target alignment across individuals. The supporting foot was positioned on a floor marker to standardize the starting position. Each kick was performed with the instruction to “execute as fast and accurately as possible,” with a 60–90 s rest period between attempts to minimize fatigue. Only the five most technically correct and target-accurate trials were selected for analysis. To facilitate kinematic tracking, visible adhesive markers of contrasting color were attached to specific anatomical landmarks, including the acromion, anterior superior iliac spine, greater trochanter, lateral femoral epicondyle, lateral malleolus, heel, and fifth metatarsal head. A visual representation of marker placement is provided in Figure 2.

These markers allowed for precise manual and semi-automatic digitization of movement using Dartfish 11 software. To enable phase-specific interpretation of the kinematic data, the execution of the Yop Chagi technique was divided into four movement phases: the Preparation Phase (from the initial ready stance to the initiation of the kicking leg lift), the Chamber Phase (characterized by hip and knee flexion prior to rapid leg extension), the Extension or Swing Phase (defined as the rapid knee extension and hip abduction until foot–target contact, representing the Impact Phase), and the Recovery Phase (from the end of contact until the kicking leg returned to the ground and postural stability was re-established). Based on these defined phases, kinematic variables included temporal parameters (total execution time, chamber phase duration, and extension phase duration), linear kinematics (maximum linear velocity and acceleration of the kicking foot, primarily during the extension phase), angular kinematics (hip, knee, and ankle joint angles and angular velocities, mainly during the chamber and extension phases), and postural control assessed by trunk inclination angle relative to the vertical axis throughout all phases. All variables were normalized to limb length to reduce anthropometric bias. For reliability, 20% of the data were independently digitized by two experienced analysts, and inter-rater reliability was assessed using intraclass correlation coefficients (ICC), with values above 0.80 considered acceptable.

### Statistical analysis

Statistical analysis was performed after testing normality using the Shapiro Wilk test. Between group comparisons (novice vs. experienced) were conducted using independent-samples t-tests for normally distributed variables and Mann Whitney U tests for non-normal distributions. Effect sizes were reported using Cohen’s d with 95% confidence intervals to quantify the magnitude of between-group differences, and their interpretation followed established guidelines (Brydges, 2019; Lakens, 2013). The significance level was set at α = 0.05 with Benjamini–Hochberg correction applied for multiple comparisons. To evaluate participants’ physical fitness, a series of standardized field tests were administered. Lower-limb explosive power was measured using the Vertical Jump test (cm), while upper-body muscular endurance was assessed through the Push-Ups test (maximum repetitions within 30 s). Core muscular endurance was evaluated using the Sit-Ups test (maximum repetitions within 30 s). Agility was measured with the Shuttle Run test (time in seconds), and sprinting ability was assessed using the 40-m Sprint test (time in seconds). All tests were conducted following standardized protocols, with participants receiving clear instructions and performing familiarization trials prior to measurement.

### Research results

**FIGURE 3:**
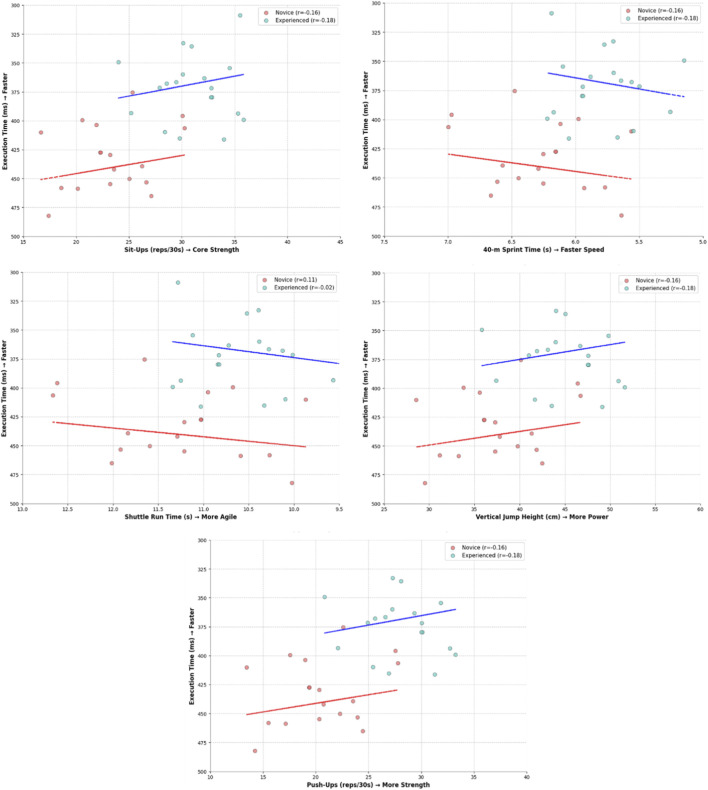
Scatter plots with regression lines showing the association of execution time with core strength (sit-ups), sprint speed (40-m sprint), agility (shuttle run), power (vertical jump), and muscular strength (push-ups) in novice and experienced groups.

The results of the fitness-related tests are presented in Table 2. Independent t-tests revealed significant differences between novice and experienced university taekwondo practitioners across all variables. In the fitness tests, the experienced group scored higher than novices in vertical jump (*p* < 0.01, Cohen’s d = 1.02), push-ups and sit-ups (*p* < 0.01, large effect sizes), shuttle run (shorter completion time; *p* < 0.01, Cohen’s d = 0.95), and 40 m sprint (*p* < 0.01). Anthropometric profiles were comparable between groups. Figure 3 illustrates the relationships between execution time and various physical fitness measures in novice and experienced groups.

**TABLE 2:** Physical performance tests (mean ± SD).

Variable	Novice (n = 20)	Experienced (n = 20)	*p*-value	Effect size (d)
Vertical jump (cm)	38.5 ± 5.2	45.7 ± 5.6	<0.01	1.02 (large)
Push-ups (reps/30s)	21.3 ± 4.1	28.6 ± 4.4	<0.01	1.70 (large)
Sit-ups (reps/30s)	24.1 ± 3.9	31.4 ± 4.2	<0.01	1.80 (large)
Shuttle run (s)	11.4 ± 0.8	10.6 ± 0.7	<0.01	0.95 (large)
40-M sprint (s)	6.35 ± 0.41	5.82 ± 0.38	<0.01	1.31 (large)

The results of the kinematic analysis are presented in Table 3. In the kinematic analysis of the side kick (Yop Chagi), experienced practitioners demonstrated greater peak hip angles (135.2° ± 7.1° vs. 118.5° ± 6.8°, *p* < 0.001, Cohen’s d = 1.65), greater peak knee angles (120.7° ± 6.5° vs. 102.3° ± 5.9°, *p* < 0.001, Cohen’s d = 1.82), higher linear velocity of the foot (9.5 ± 0.8 vs. 6.8 ± 0.7 m/s, *p* < 0.001, Cohen’s d = 2.46), higher angular velocity of the knee (1,065.7 ± 82.1 vs. 820.4° ± 75.3°/s, *p* < 0.001, Cohen’s d = 2.14), and shorter execution time (368.9 ± 30.2 vs. 450.6 ± 38.4 m, *p* < 0.001, Cohen’s d = 1.69) compared to novices.

**TABLE 3:** Biomechanical variables analyzed: peak hip angle, peak knee angle, foot linear velocity, knee angular velocity, and execution time.

Variable	Novice (n = 20) Mean ± SD	Experienced (n = 20) Mean ± SD	t/U value	*p*-value	Effect size (Cohen’s d)
Peak hip Angle (°)	118.5 ± 6.8	135.2 ± 7.1	t = −7.21	<0.001	1.65 (large)
Peak knee Angle (°)	102.3 ± 5.9	120.7 ± 6.5	t = −8.14	<0.001	1.82 (large)
Linear velocity of foot (m/s)	6.8 ± 0.7	9.5 ± 0.8	t = −11.02	<0.001	2.46 (very large)
Angular velocity of knee (°/s)	820.4 ± 75.3	1,065.7 ± 82.1	t = −9.61	<0.001	2.14 (very large)
Execution Time (ms)	450.6 ± 38.4	368.9 ± 30.2	t = 7.29	<0.001	1.69 (large)

## Discussion

The present study provides a kinematic comparison of the Taekwondo side kick (*Yop Chagi*) between novice and experienced university practitioners. Experienced athletes demonstrated more efficient kicking mechanics, with larger joint angles, higher angular and linear velocities, and shorter execution times than novices (Huang et al., 2025; Jia et al., 2023; Vagner et al., 2023). In novices, faster execution times were weakly associated with general physical fitness components such as core strength, sprint speed, agility, and muscular strength (Sant’Ana et al., 2017; Zhang et al., 2025) Conversely, experienced practitioners showed stronger negative relationships between execution time and sprint speed and agility, suggesting these factors are more decisive in skilled performance (Arabacı et al., 2010; Gisladottir et al., 2024). Higher core strength and muscular power did not always correspond to shorter execution times in experienced athletes, indicating that motor skill proficiency mediates the translation of physical fitness into technical performance (Guan et al., 2024; Zhang et al., 2025).

Interpretation of key kinematic differences. Experienced practitioners demonstrated greater hip and knee angles at peak kicking, reflecting both enhanced ROM and refined motor control (Falco et al., 2011; Sørensen et al., 1996). This larger chambering allows more efficient limb acceleration and higher foot velocity, consistent with the kinetic chain principle and elastic energy transfer during extension (Almansoof et al., 2023; Sant’ Ana et al., 2021). Higher angular and linear velocities are supported by long-term neuromuscular adaptations, including improved inter-muscular coordination, selective fast-twitch fiber recruitment, and reduced antagonist co-contraction (Aslam et al., 2025; Kim et al., 2010). The resulting shorter execution time provides a clear tactical advantage in both poomsae and kyorugi (Kunay et al., 2025; Podrigalo et al., 2025). These kinematic efficiencies are underpinned by superior physical fitness (vertical jump, endurance, agility, speed), although performance is mediated by motor skill proficiency rather than strength alone, as evidenced by the correlation between jump height and kick speed (Karine et al., 2016; Martínez-Majolero et al., 2013; Ouergui et al., 2025). Overall, these findings suggest that in skilled athletes, technical execution is optimized through the integration of physical capacity and coordinated motor control.

Proximal-to-distal sequencing and coordination. Experienced athletes excel in inter-segmental coordination, particularly the proximal-to-distal sequence, which begins at the pelvis and torso, progresses through the thigh, and culminates at the foot, allowing successive segments to reach peak velocity just before impact (Falco et al., 2011; Kwan Kim et al., 2011). This sequencing enhances speed summation and impact efficiency. Skilled Taekwondo athletes show precise temporal coupling between segments, maximizing foot velocity and force (Estevan et al., 2011; Estevan et al., 2012), whereas novices exhibit less coordinated, segment-dominant patterns, causing energy loss and lower final foot speed. Studies comparing elite and non-elite practitioners confirm that better proximal-to-distal coordination correlates with superior kicking performance and technical efficiency (Reum Hong and Moo So, 2012). These results indicate that in skilled athletes, motor skill proficiency, not just joint motion or strength, mediates the translation of physical fitness into effective technical output.

Practical applications and implications. The findings provide practical guidance for coaches and educators. For technique training, simple repetition is insufficient; emphasis should be on the movement sequence initiating with the hip, driving the knee, and snapping the foot using drills that isolate the chamber and explosive extension phases (Falco et al., 2011; Kwan Kim et al., 2011). Physical conditioning should target lower-body explosive power through plyometrics and Olympic lifts, as well as core stability, to support the neuromuscular demands of skilled kicking (Estevan et al., 2012). For novice pedagogy, instructors should teach fundamental biomechanical principles and use visual feedback via video analysis (e.g., Dartfish) to help learners cognitively understand optimal movement patterns (Barbieri et al., 2010; Falco et al., 2011). These strategies integrate technical, physical, and cognitive elements to enhance skill acquisition and performance efficiency in Taekwondo athletes.

This study has several limitations. First, the two-dimensional kinematic analysis, while reliable for sagittal plane motion, cannot capture movements in the transverse and frontal planes, such as pelvic rotation or hip abduction/adduction, which are also relevant to Yop Chagi. Future research should employ three-dimensional motion capture to provide a more complete analysis. Second, the study was limited to kinematic variables. Integrating kinetics (e.g., force plate data to measure ground reaction forces) and electromyography (EMG) to assess muscle activation patterns would offer a deeper understanding of the mechanisms driving the observed kinematic differences. Finally, the cross-sectional design compares different individuals. Longitudinal studies tracking the same cohort of novices over an extended training period would provide stronger evidence for causal relationships between training, biomechanical adaptation, and performance enhancement.

## Conclusion

This study demonstrates that experienced university Taekwondo practitioners perform the Yop Chagi with significantly greater kinematic efficiency than novices, as evidenced by higher joint velocities, faster execution, and optimized inter-segmental coordination. These results indicate that effective kicking performance depends not only on physical fitness but also on neuromuscular coordination developed through systematic, long-term practice. Practically, training programs should integrate exercises targeting both strength and coordination to enhance kicking technique, providing coaches and educators with evidence based guidance for skill development.

## Data Availability

The original contributions presented in the study are included in the article/supplementary material, further inquiries can be directed to the corresponding author.
